# Dickkopf2 rescues erectile function by enhancing penile neurovascular regeneration in a mouse model of cavernous nerve injury

**DOI:** 10.1038/s41598-017-17862-5

**Published:** 2017-12-19

**Authors:** Kalyan Ghatak, Guo Nan Yin, Min-Ji Choi, Anita Limanjaya, Nguyen Nhat Minh, Jiyeon Ock, Kang-Moon Song, Dong Hyuk Kang, Young-Guen Kwon, Ho Min Kim, Ji-Kan Ryu, Jun-Kyu Suh

**Affiliations:** 1 National Research Center for Sexual Medicine and Department of Urology, Inha University School of Medicine, Incheon, 22332 Republic of Korea; 2 Department of Biochemistry, College of Life Science and Biotechnology, Yonsei University, Seoul, 03722 Republic of Korea; 3 Graduate School of Medical Science and Engineering, Korea Advanced Institute of Science and Technology (KAIST), Daejeon, 34141 Republic of Korea; 4 Inha Research Institute for Medical Sciences, Inha University College of Medicine, Incheon, 22212 Republic of Korea

## Abstract

Penile erection is a neurovascular event and neurologic or vascular disturbances are major causes of erectile dysfunction (ED). Radical prostatectomy for prostate cancer not only induces cavernous nerve injury (CNI) but also results in cavernous angiopathy, which is responsible for poor responsiveness to oral phosphodiesterase-5 inhibitors. Dickkopf2 (DKK2) is known as a Wnt signaling antagonist and is reported to promote mature and stable blood vessel formation. Here, we demonstrated in CNI mice that overexpression of DKK2 by administering DKK2 protein or by using DKK2-Tg mice successfully restored erectile function: this recovery was accompanied by enhanced neural regeneration through the secretion of neurotrophic factors, and restoration of cavernous endothelial cell and pericyte content. DKK2 protein also promoted neurite outgrowth in an *ex vivo* major pelvic ganglion culture experiment and enhanced tube formation in primary cultured mouse cavernous endothelial cells and pericytes co-culture system *in vitro*. In light of critical role of neuropathy and angiopathy in the pathogenesis of radical prostatectomy-induced ED, reprogramming of damaged erectile tissue toward neurovascular repair by use of a DKK2 therapeutic protein may represent viable treatment option for this condition.

## Introduction

Penile erection is a neurovascular event characterized by tumescence of the cavernous bodies that relies upon functional interaction between neuronal and vascular components^1,2^. Functional and structural impairments of neuronal cells, endothelial cells, and pericytes are involved in the pathogenesis of erectile dysfunction (ED)^1,2^, a condition defined as an inability to attain or maintain penile erection sufficient for satisfactory sexual intercourse^3^. ED is one of the most common complications following radical prostatectomy for prostate cancer, which is highly prevalent in elderly male papulation^4^. Even with introduction of robotic procedures and bilateral nerve sparing approaches, partial cavernous nerve injury (CNI) or neurapraxia is unavoidable^5,6^. Long-term denervation of erectile tissue leads to cavernous hypoxia and structural changes in erectile tissue, including increase in cavernous endothelial and smooth muscle cell apoptosis, and cavernous fibrosis from collagen deposition^7–10^, which result in decreased responsiveness to oral phosphodiesterase-5 (PDE5) inhibitors^11,12^. Therefore, new treatment strategies that correct cavernous nerve damage as well as vascular dysfunction are necessary for patients with radical prostatectomy-induced ED to overcome the limitation of PDE5 inhibitors.

Advances in the science in the neurobiology field have led us to neuromodulatory strategy, including immunophilin ligands and neurotrophic factors, to protect or regenerate damaged cavernous nerve^13^. Although immunophilin ligands have been shown to induce recovery of erectile function at the preclinical level^13^, a clinical trial in men radical prostatectomy-induced ED failed to show the recovery of erectile function^14^. This finding may imply that functional and structural derangements in erectile tissue, such as endothelial cell apoptosis and fibrosis, have already begun after denervation. Therefore, it would be ideal to develop a treatment modality targeting both neural regeneration and angiogenesis.

Wnt signaling pathway is known to be involved in a wide range of cellular and biological processes, such as cell proliferation, survival, polarity, adhesion, and migration^15^. The dickkopf-related families (DKK1-DKK4) are secreted proteins and functionally act as either an agonist or antagonist of Wnt signaling by binding to low density lipoprotein (LRP) receptor-related protein 5/6^16^. Dickkopf-2 (DKK2), known as a Wnt signaling antagonist, has been reported to enhance endothelial cell migration independent of Wnt and promote neovascularization in animal models of hind limb ischemia and myocardial infarction^17^. Furthermore, DKK2 induced mature and stable blood vessel formation in a corneal angiogenesis assay by enhancing pericyte coverage on endothelial cells. And consequently, DKK2-induced capillaries were less leaky than vascular endothelial growth factor (VEGF)-induced vessels^17^. However, the effect of DKK2 on nervous system and erectile function has not yet been explored.

In the present study, we examined the effectiveness and mechanism through which DKK2 restores CNI-induced ED by local administration of DKK2 protein into the penis or by overexpression of DKK2 by using DKK2-transgenic (DKK2-Tg) mice under the control of the endothelial cell-specific Tie2 promoter/enhancer. We also determined the neurotrophic and angiogenic potential of DKK2 protein in an *ex vivo* major pelvic ganglion (MPG) culture experiment and in primary cultured mouse cavernous endothelial cell (MCEC) and pericyte (MCP) mono-culture or co-culture system *in vitro*.

## Results

### DKK2 restores erectile function in CNI mice

**Figure 1:**
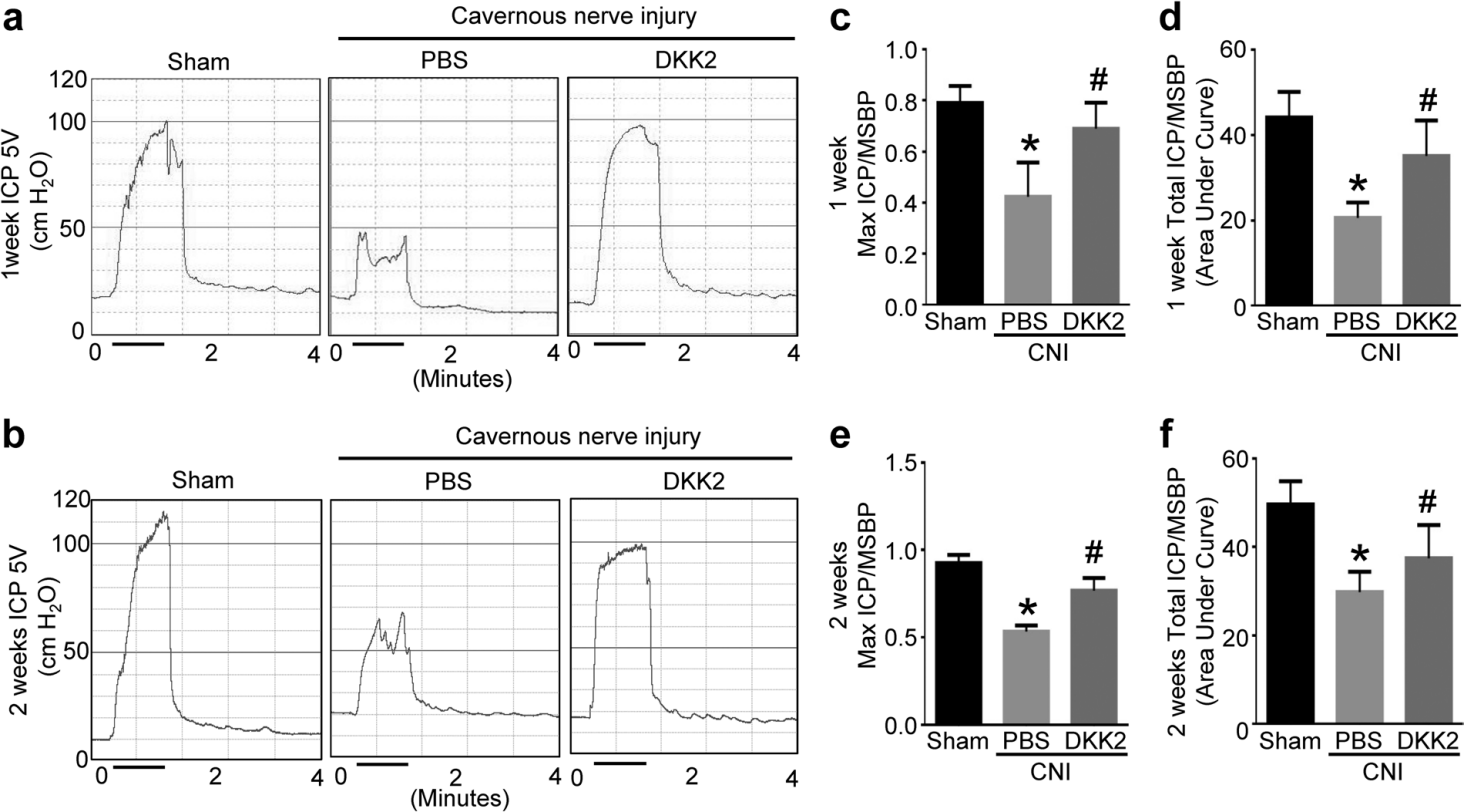
DKK2 restores intracavernous pressure (ICP) elicited by electrical stimulation of the cavernous nerve. (**a**,**b**) Representative ICP responses for the sham operation group or CNI mice stimulated at 1 or 2 weeks after intracavernous injections of PBS (20 μl) or DKK2 protein (days -3 and 0; 6 µg/20 µl). The stimulus interval is indicated by a solid bar. **(c**–**f)** Ratios of mean maximal ICP and total ICP (area under the curve) to mean systolic blood pressure (MSBP) were calculated for each group. Each bar depicts the mean (±SE) values from n = 6 animals per group. *P < 0.001 vs. sham operation group, ^#^ *P* < 0.05 vs. PBS-treated CNI group. CNI = cavernous nerve injury; DKK2 = dickkopf2.

To determine the physiological consequence of intracavernous injections of DKK2 protein, we evaluated erectile function during electrical stimulation of the cavernous nerve *in vivo*. A representative intracavernous tracing after stimulation of the cavernous nerve (5 V, 12 Hz, 1 msec) for 1 min in sham control or CNI mice 1 and 2 weeks after treatment is shown in Fig. 1a,b. The ratios of maximal intracavernous pressure (ICP) and total ICP to mean systolic blood pressure (MSBP) were significantly lower in PBS-treated CNI mice than in the sham group. Intracavernous injections of DKK2 protein (days -3 and 0; 6 µg/20 µl) induced profound restoration of erectile function at 1 week after treatment (90% of sham control values), and resulted in partial recovery of erectile function at 2 weeks (Fig. 1c–f). No detectable differences were found in MSBP and body weight among the experimental groups (see Supplementary information Table S1).

### DKK2 promotes neural regeneration through enhanced production of neurotrophic factors in CNI mice *in vivo* and in MPG culture *in vitro*

**Figure 2:**
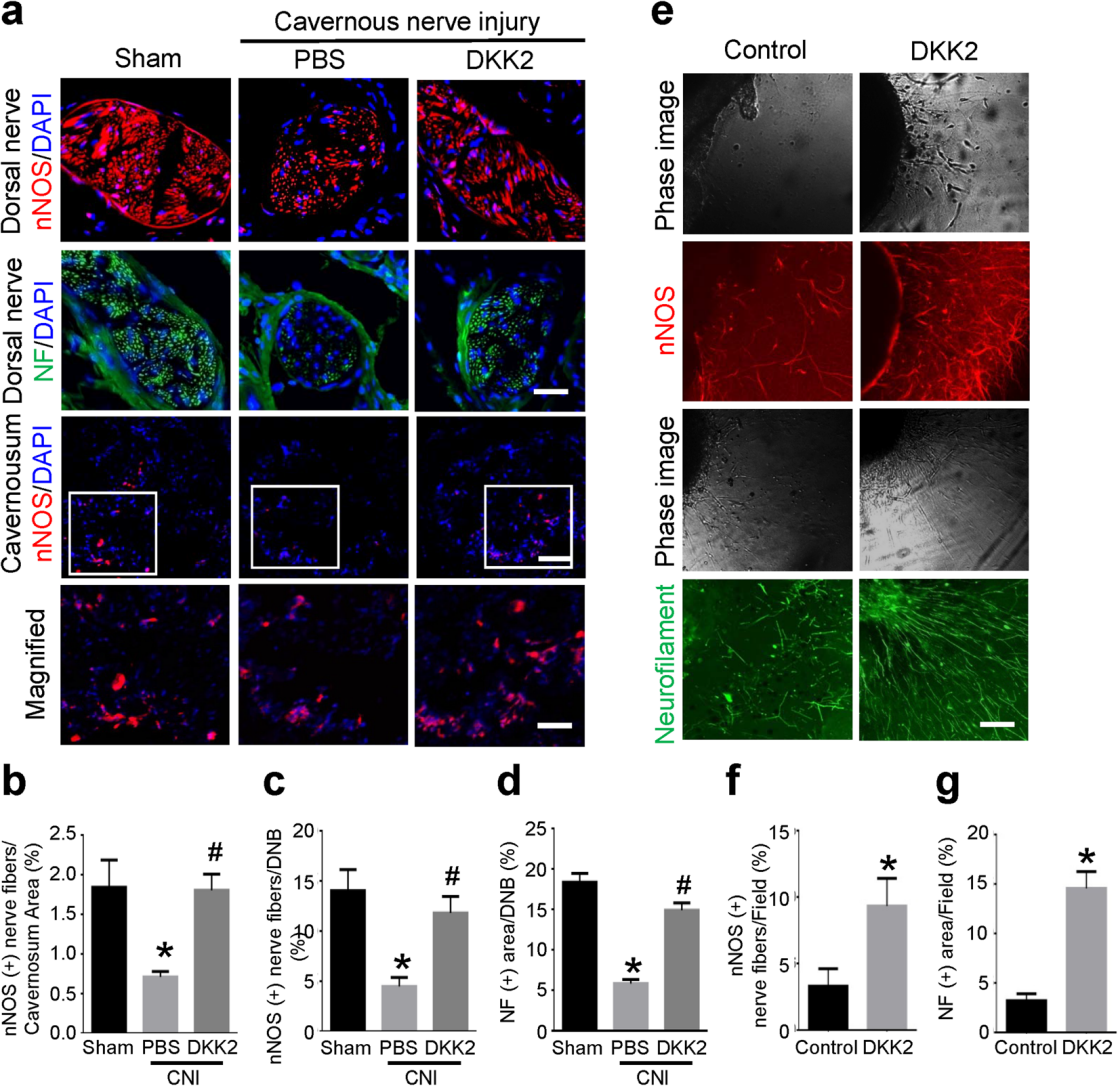
DKK2 induces neural regeneration. **(a)** nNOS (red) and neurofilament (NF, green) staining of penis tissue from sham operation group or CNI mice 1 week after receiving intracavernous injections of PBS (20 μl) or DKK2 protein (days -3 and 0; 6 µg/20 µl). Nuclei were labeled with DAPI (blue). Scale bar 25 µm (upper row), 100 µm (mid row), and 25 µm (lower row). **(b–d)** Quantitative analysis of cavernous nNOS- and neurofilament-immunopositive area in cavernous tissue or dorsal nerve bundle (DNB) was performed by an image analyzer. Each bar depicts the mean ( ± SE) values from n = 6 animals per group. *P < 0.05 vs. sham operation group, ^#^ *P* < 0.05 vs. PBS-treated CNI group. **(e)** Neurofilament (green) and nNOS (red) staining in mouse major pelvic ganglion (MPG) tissue, which were treated with PBS or DKK2 protein (300 ng/ml). Scale bar = 200 μm. **(f**,**g)** Quantitative analysis of neurofilament- or nNOS-immunopositive neurite length was performed by an image analyzer. Each bar depicts the mean (±SE) values from n = 4 independent experiments. *P < 0.001 vs. control group. CNI = cavernous nerve injury; DKK2 = dickkopf2; nNOS, neuronal nitric oxide synthase.

The expression of neuronal nitric oxide synthase (nNOS) and neurofilament in the dorsal nerve bundle or the corpus cavernosum tissue was significantly lower in the PBS-treated CNI group than in the sham group. Intracavernous administration of DKK2 protein significantly restored penile nNOS-containing fiber and axonal contents (neurofilament) in CNI mice (Fig. 2a–d). To test neurotrophic effect of DKK2 *in vitro*, we isolated and cultivated mouse MPG in matrigel. The MPG tissues were then treated with to PBS or DKK2 protein (300 ng/ml). At 5 days after incubation, immunohistochemical staining of MPG tissue with antibodies to neurofilament and nNOS showed that the neurite sprouting was significantly higher in MPG tissue treated with DKK2 protein than in the tissue treated with PBS (Fig. 2e–g).

**Figure 3:**
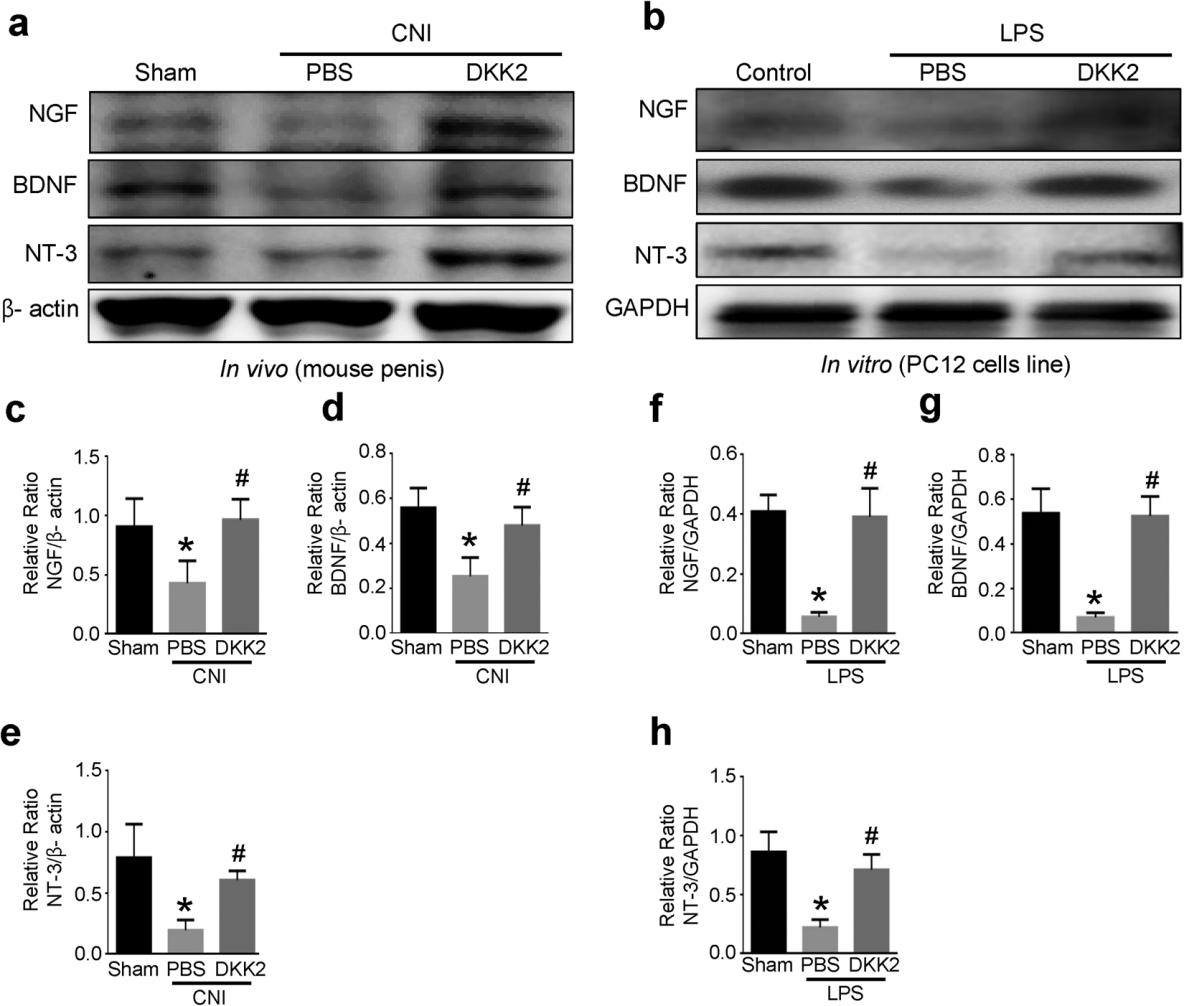
DKK2 increases the expression of neurotrophic factors. (**a**,**b)** Representative Western blot for neurotrophic factors (NGF, BDNF, and NT-3) in penis tissue from sham operation group or CNI mice 1 week after receiving intracavernous injections of PBS (20 μl) or DKK2 protein (days -3 and 0; 6 µg/20 µl); and in PC12 cells exposed to lipopolysaccharide (LPS), which were treated with DKK2 protein (300 ng/ml). **(c–h)** Data are presented as the relative density of each protein compared with that of β-actin or GAPDH. Each bar depicts the mean (±SE) values from n = 4 independent samples. *P < 0.05 vs. sham operation group, ^#^ *P* < 0.05 vs. PBS-treated group. BDNF = brain-derived neurotrophic factor; CNI = cavernous nerve injury; DKK2 = dickkopf2; NGF = nerve growth factor; NT-3 = neurotrophin-3. All the blots/gels were presented by using cropped images (Full-length blots/gels images are presented in Supplementary Figure S1).

Next, we asked whether the neurotrophic effects of DKK2 were mediated by the production of neurotrophic factors. The expression of nerve growth factor (NGF), brain-derived neurotrophic factor (BDNF), and neurotrophin-3 (NT-3) was significantly lower in the PBS-treated CNI group and in lipopolysaccharide (LPS)-treated PC12 cells than in the sham operation group and in untreated PC12 cells, respectively. DKK2 protein significantly enhanced expression of the neurotrophic factors in both CNI mice *in vivo* and LPS-treated PC12 cells *in vitro* (Fig. 3).

### DKK2 restores cavernous endothelial and pericyte content in CNI mice

**Figure 4:**
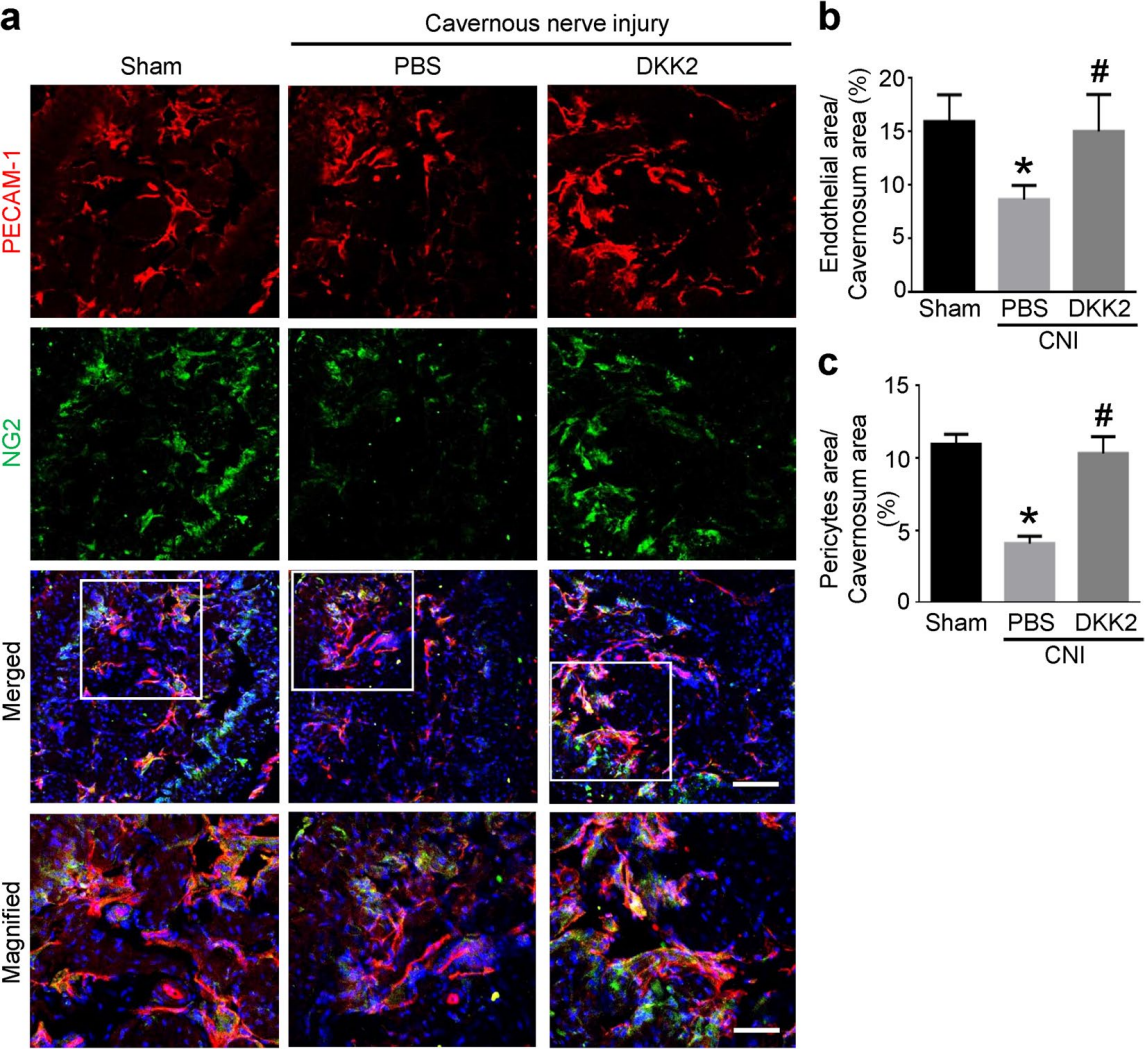
DKK2 restores cavernous endothelial and pericyte content. **(a)** PECAM-1 (red) and NG2 (green) staining of cavernous tissue from sham operation group or CNI mice 1 week after receiving intracavernous injections of PBS (20 μl) or DKK2 protein (days -3 and 0; 6 µg/20 µl). Nuclei were labeled with DAPI (blue). Scale bar 100 µm (upper row), and 25 µm (lower row). **(b**,**c)** Quantitative analysis of cavernous endothelial cell and pericyte content was performed by an image analyzer. Each bar depicts the mean (±SE) values from n = 6 animals per group. *P < 0.05 vs. sham operation group, ^#^ *P* < 0.05 vs. PBS-treated CNI group. CNI = cavernous nerve injury; DKK2 = dickkopf2.

Immunofluorescent staining of cavernous tissue with antibodies to PECAM-1 and NG2 was performed in sham control or CNI mice 1 week after treatment. We found significantly lower cavernous endothelial and pericyte content in the PBS-treated CNI mice than in the sham group. Cavernous endothelial and pericyte content was completely restored in CNI mice treated with DKK2 protein (Fig. 4).

### DKK2 decreases cavernous endothelial cell apoptosis and promotes endothelial proliferation in CNI mice

**Figure 5:**
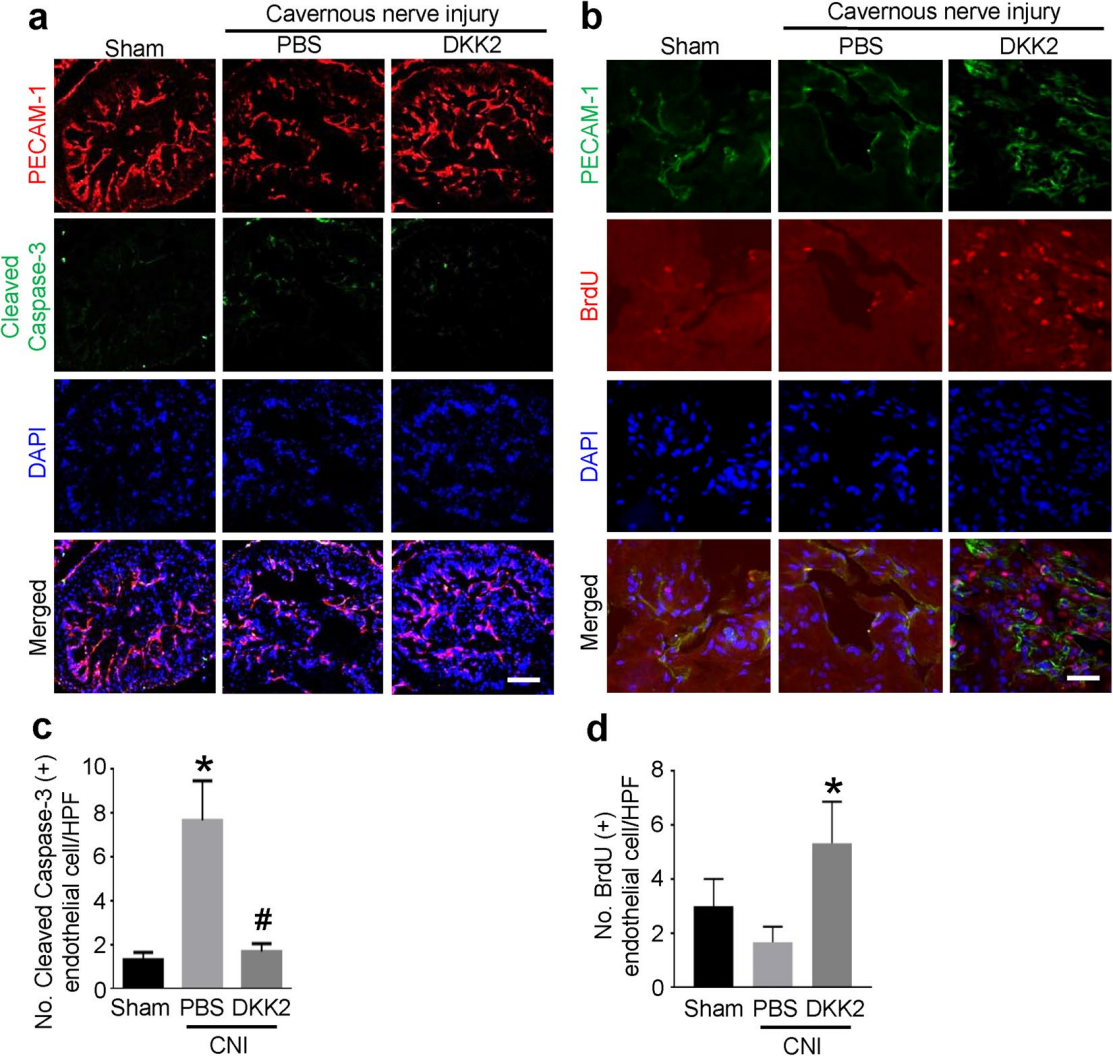
DKK2 decreases cavernous endothelial cell apoptosis and enhances endothelial cell proliferation. (**a**,**b**) PECAM-1 (red) and cleaved caspase-3 (green) or PECAM-1 (green) and BrdU (red) staining of cavernous tissue from sham operation group or CNI mice 1 week after receiving intracavernous injections of PBS (20 μl) or DKK2 protein (days -3 and 0; 6 µg/20 µl). Nuclei were labeled with DAPI (blue). Scale bar 100 µm (left row), and 25 µm (right row). **(c)** Number of apoptotic cells in endothelial cells per high-power field (screen magnification ×200). Each bar depicts the mean (±SE) values from n = 6 animals per group. *P < 0.001 vs. sham operation group, ^#^ *P* < 0.001 vs. PBS group. **(d)** Number of BrdU-immunopositive endothelial cells per high-power field (screen magnification ×400). Each bar depicts the mean (±SE) values from n = 4 animals per group. *P < 0.01 vs. PBS-treated CNI group. CNI = cavernous nerve injury; DKK2 = dickkopf2.

Dual labeling of cavernous tissue with antibodies to PECAM-1 and cleaved capase-3 showed that the number of apoptotic cells in cavernous endothelial cells was significantly greater in the PBS-treated CNI group than in the sham group. Intracavernous injections of DKK2 protein significantly decreased apoptosis in the cavernous endothelial cells of the CNI mice, which was comparable to the level found in the sham group (Fig. 5a,c).

To test whether the DKK2-induced increase in cavernous endothelial content resulted from endothelial proliferation, we assessed the number of endothelial cells positive for BrdU. We observed significant increase in BrdU-positive endothelial cells in CNI mice treated with DKK2 protein (Fig. 5b,d).

### DKK2 promotes tube formation in primary cultured MCEC and MCP *in vitro*

**Figure 6:**
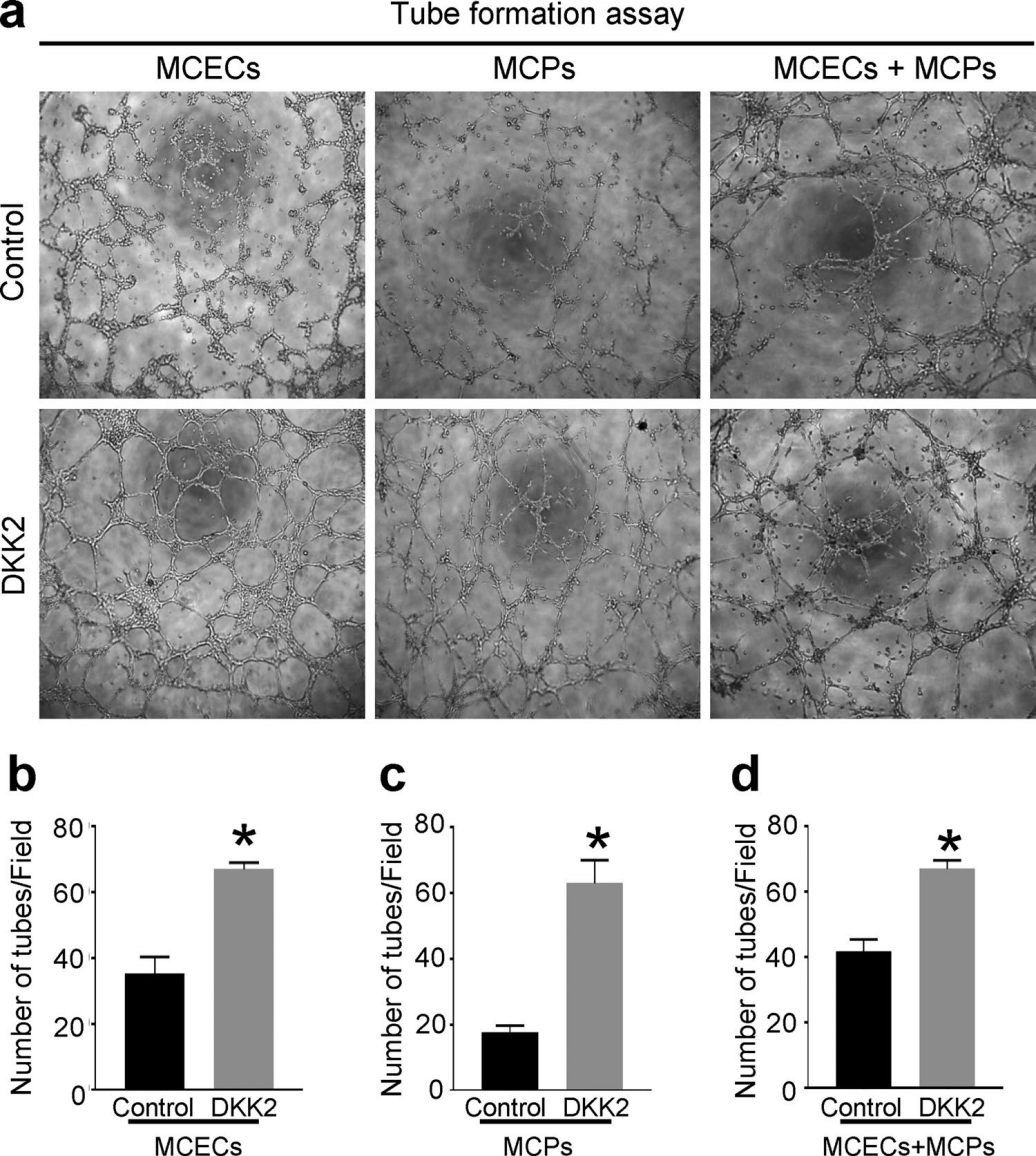
DKK2 enhances tube formation. **(a)** Tube formation assay in primary cultured mouse cavernous endothelial cells (MCEC) and pericytes (MCP) mono-culture system or in MCEC-MCP co-culture system, which were treated with PBS or DKK2 protein (300 ng/ml). **(b–d)** Number of tubes per high-power field. Each bar depicts the mean (±SE) values from n = 4 independent experiments. *P < 0.001 vs. control group. DKK2 = dickkopf2.

We further examined the role of DKK2 in MCEC and MCP mono-culture or direct mixed co-culture system. The primary cultured MCEC and MCP were serum-starved for 24 hours and were exposed to PBS or DKK2 protein (300 ng/ml) for 48 hours. *In vitro* matrigel assay revealed that treatment of the cells with DKK2 protein profoundly enhanced tube formation in both endothelial cell-pericyte mono-culture and co-culture system. MCEC and MCP mono-culture or the mixture of these cells formed well-organized capillary-like structures by treatment with DKK2 protein (Fig. 6).

### DKK2-Tg mice are resistant to CNI-induced neuropathy and angiopathy and have restored erectile function

**Figure 7:**
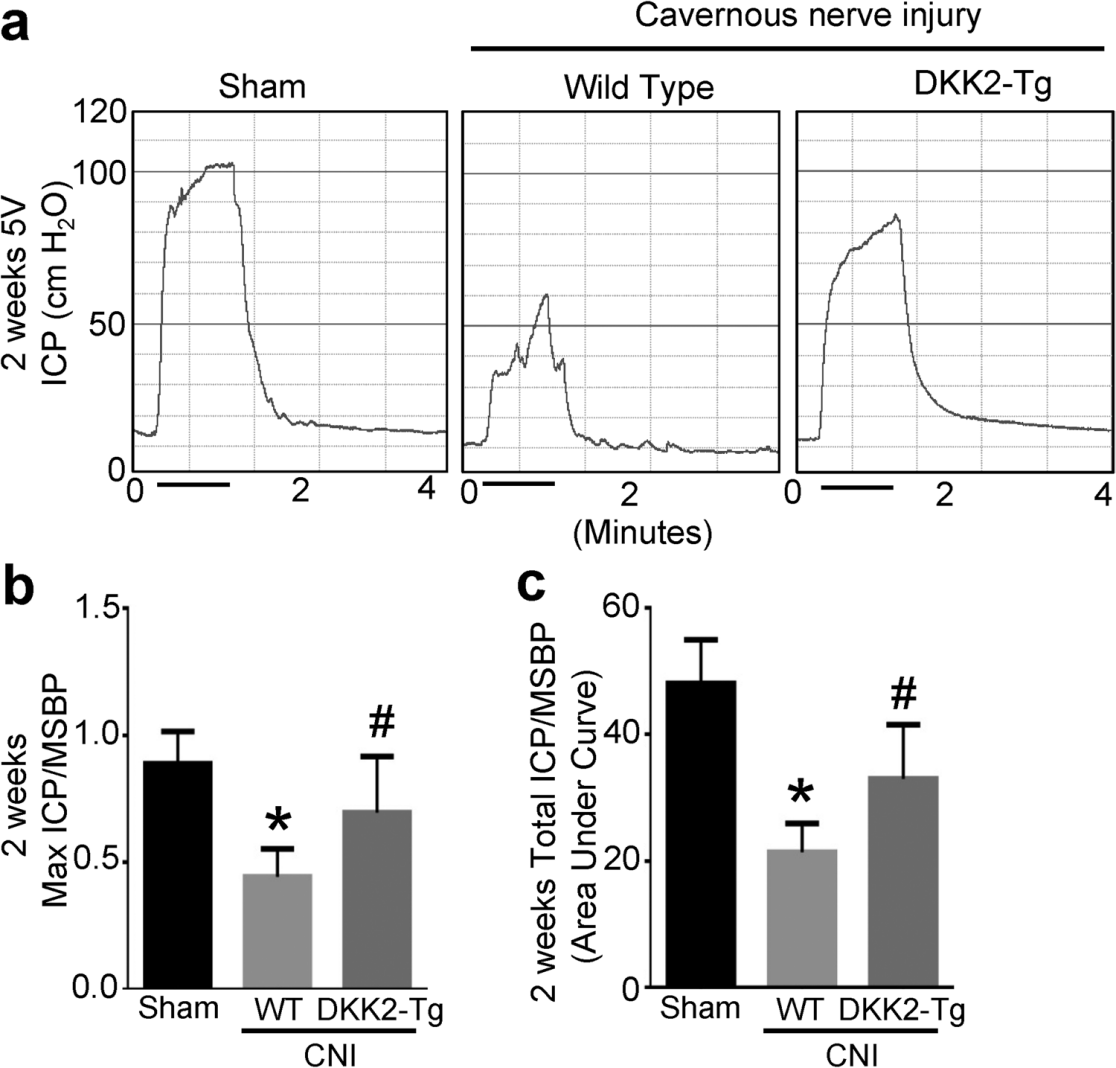
DKK2-Tg mice are resistant to CNI-induced erectile dysfunction. (**a)** Representative ICP responses for the wild-type (WT) mice receiving sham operation, WT mice receiving CNI, or DKK2-Tg mice receiving CNI. The stimulus interval is indicated by a solid bar. The ICP was measured 2 weeks after CNI. **(b**,**c)** Ratios of mean maximal ICP and total ICP (area under the curve) to mean systolic blood pressure (MSBP) were calculated for each group. Each bar depicts the mean (±SE) values from n = 6 animals per group. *P < 0.001 vs. WT + sham operation group, ^#^ *P* < 0.05 vs. WT + CNI group. CNI = cavernous nerve injury; DKK2-Tg = dickkopf2-transgenic mice.

To further confirm the above results regarding the role of DKK2 in the restoration of erectile function in CNI mice, we examined the effect of CNI in DKK2-Tg mice. At 2 weeks after CNI, the ratios of maximal ICP and total ICP to MSBP were significantly lower in wild-type (WT) that received CNI than in sham-operated WT mice, whereas erectile function was significantly preserved in DKK2-Tg mice that received CNI (Fig. 7). The body weight and MSBP were did not differ significantly among the experimental groups (see Supplementary information Table S2).

**Figure 8:**
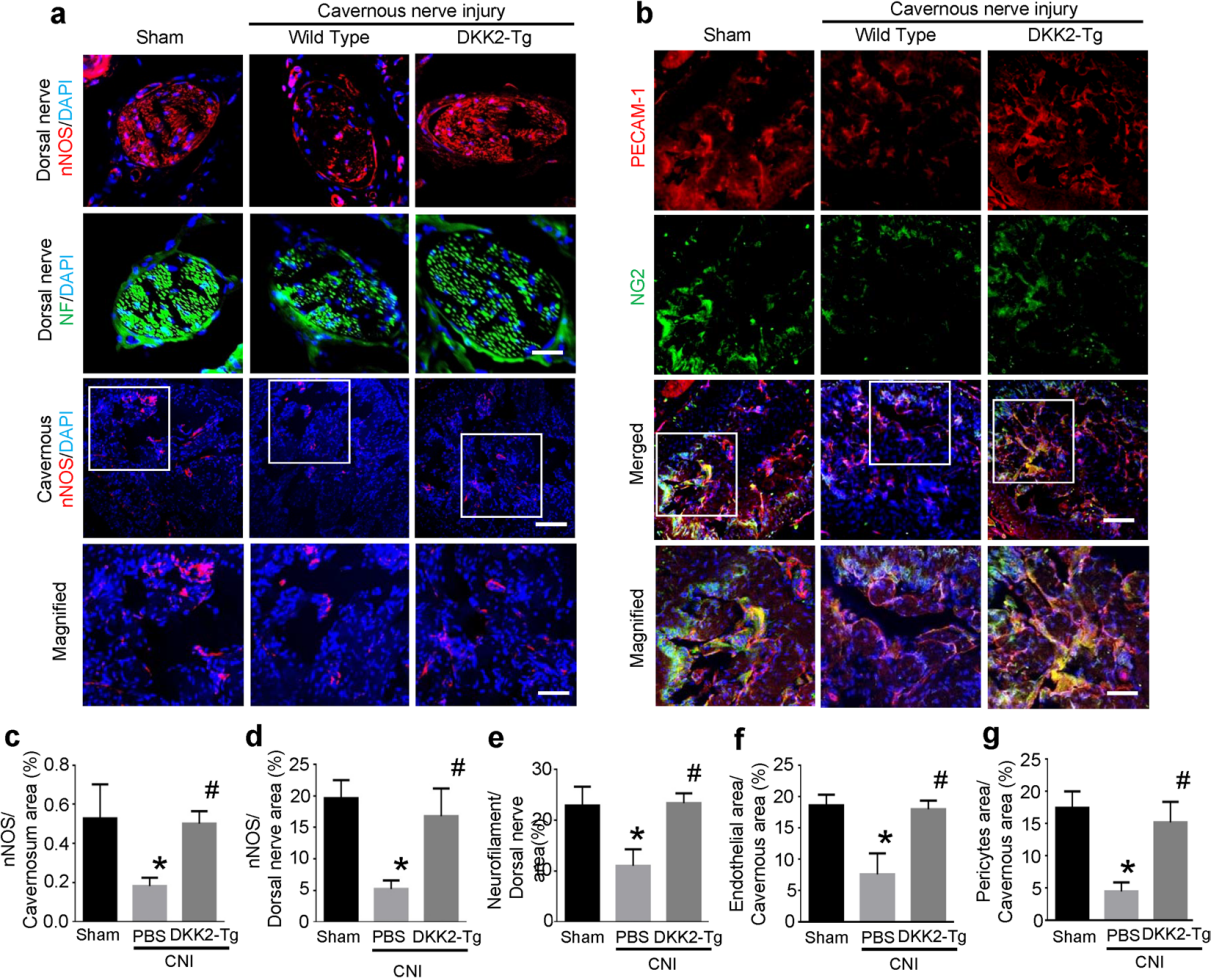
DKK2-Tg mice are resistant to CNI-induced neuropathy and angiopathy. (**a)** nNOS (red) and neurofilament (NF, green) staining of penis tissue from wild-type (WT) mice receiving sham operation, WT mice receiving CNI, or DKK2-Tg mice receiving CNI. Nuclei were labeled with DAPI (blue). Scale bar 25 µm (upper row), 100 µm (mid row), and 25 µm (lower row). **(b)** PECAM-1 (red) and NG2 (green) staining of cavernous tissue from each group. Scale bar 100 µm (upper row), and 25 µm (lower row). **(c–g)** Quantitative analysis of nNOS- and nerofilament-immunopositive area in dorsal nerve bundle, and cavernous endothelial and pericyte content was performed by an image analyzer. Each bar depicts the mean ( ± SE) values from n = 4 animals per group. *P < 0.001 vs. WT + sham operation group, ^#^ *P* < 0.001 vs. WT + CNI group. CNI = cavernous nerve injury; DKK2-Tg = dickkopf2-transgenic mice.

In accordance with this finding, overexpression of DKK2 gene rescued the CNI-induced decrease in neuronal cell contents as well as cavernous endothelial and pericyte contents (Fig. 8).

## Discussion

Here, we investigated whether overexpression of DKK2 exerts beneficial effects in CNI-induced ED. Our results showed that overexpression of DKK2 by administering DKK2 protein or by using DKK2-Tg mice successfully restored erectile function: this recovery was accompanied by enhanced penile neural regeneration through the secretion of neurotrophic factors, restoration of cavernous endothelial cell and pericyte content, and decreased endothelial cell apoptosis and enhanced endothelial cell proliferation. DKK2 protein also restored expression of the neurotrophic factors in LPS-treated PC12 cells *in vitro* and promoted neurite outgrowth in an *ex vivo* MPG culture. Moreover, DKK2 protein accelerated tube formation in primary cultured MCEC and MCP mono-culture or co-culture system *in vitro*.

The cavernous nerve carries nNOS-positive nerve fibers and generates survival signals to erectile tissue. The amount of intact nNOS-positive neurons is important for proper erectile function. Nitric oxide derived from nerve terminals initiates penile erection through dilation of the cavernous artery and sinusoids. Subsequent shear stress sustains penile erection through activation of Akt and endothelial NOS^18^. Similar to the results from previous studies^19,20^, the expression of nNOS and neurofilament in the dorsal nerve bundle or the corpus cavernosum tissue was significantly decreased after CNI. In the present study, intracavernous delivery of DKK2 protein significantly restored nNOS and neurofilament expression in the penis of CNI mice, and promoted neurite sprouting from MPG. PC12 cells were treated with LPS to mimic *in vivo* neuroinflammatory condition of CNI following radical prostatectomy. We observed in the penis of CNI mice or in PC12 cells exposed to LPS that the expression of NGF, BDNF, and NT-3 restored remarkably by treatment with DKK2 protein. It has been reported that the activation of Wnt signal is involved in neuroprotection^21,22^. Therefore, we can speculate that DKK2, a Wnt signaling antagonist, may exert its neurotrophic effects independent of Wnt pathway. To the best of our knowledge, this is the first study to document neurotrophic effects of DKK2. However, the exact mechanism by which DKK2 regulates the expression of neurotrophic factors remains to be elucidated.

Similar to the results of previous studies showing a decrease in cavernous endothelial content after CNI^23–25^, in the present study, a significant decrease in cavernous endothelial area was noted in PBS-treated CNI mice compared with the sham group. Intracavernous administration of DKK2 protein completely restored cavernous endothelial content in the CNI mice. Immunochemical staining of cavernous tissue with antibody to cleaved caspase-3 or BrdU showed that DKK2 protein decreases cavernous endothelial cell apoptosis and promotes endothelial cell proliferation. DKK2 also enhanced tube formation in primary cultured MCEC. Therefore, we can speculate that DKK2 restores cavernous endothelial cell content by inhibiting endothelial cell apoptosis and by promoting endothelial cell proliferation.

Pericytes are known to be involved in the maturation of the blood vessel and the regulation of blood flow as well as vascular permeability. Pericytes are also regarded as a potential source of endogenous mesenchymal stem cells^26–28^. Moreover, the interaction between endothelial cells and pericytes plays a crucial role in the blood vessel formation and vascular maturation^28^. We recently for the first time documented the presence of the pericytes in the cavernous sinusoids and microvessels of erectile tissue in mice or human by using immunohistochemistry. The presence of pericytes was further confirmed by primary isolation and cultivation of pericytes from erectile tissue^2^. Similar to the results from previous study showing enhanced pericyte coverage on endothelial cells by DKK2 in a corneal angiogenesis assay^17^, DKK2 completely restored cavernous pericyte content in CNI mice *in vivo* and enhanced tube formation in primary cultured MCP *in vitro*. We also established MCEC-MCP co-culture system to study cellular interaction between endothelial cells and pericytes^29^. DKK2 protein enhanced tube formation and formed well-organized capillary-like structures in MCEC-MCP co-culture.

Despite promising preclinical results of the VEGF gene or protein in a variety of animal models of ED^30–32^, its clinical application is greatly limited because VEGF often produces leaky, inflamed, and immature blood vessels^33^. In contrast, the results from previous study^17^ and the present study indicate that DKK2 promotes more stable and mature blood vessel formation than does VEGF.

Finally, we further examined the effect of the overexpression of DKK2 gene on CNI-induced neuropathy and angiopathy. DKK2-Tg mice that received CNI showed preservation of neuronal cell contents as well as cavernous endothelial and pericyte contents. In agreement with these findings, overexpression of DKK2 gene rescued the CNI-induced deterioration of erectile function.

Our study has some limitations. First, we did not perform a dose-dependent erectile function experiment for DKK protein. Second, we administered DKK2 protein at the time of CNI and provided relatively short-term efficacy data. Therefore, further studies are needed to test whether immediate or delayed administration of DKK2 protein after CNI induces long-lasting recovery of erectile function.

In summary, our results indicate that DKK2 rescues erectile function in CNI mice by inducing both neural regeneration and angiogenesis. In light of critical role of neuropathy and angiopathy in the pathogenesis of radical prostatectomy-induced ED, reprogramming of damaged erectile tissue toward neurovascular repair by use of a DKK2 therapeutic protein may represent viable treatment option for this condition.

## Methods

Methods details are given in the Supplementary information.

### Study design

The primary aim of the present study was to investigate the mechanisms and the effectiveness through which DKK2 restores CNI-induced ED. To do so, we administered DKK2 protein into the penis of WT mice with CNI and also used DKK2-Tg mice that received CNI. Detailed mechanisms were evaluated in WT and DKK2-Tg mice *in vivo*; in an *ex vivo* MPG tissue culture; and in primary cultured MCEC, MCP, and PC12 cells *in vitro*.

### Animals and treatments

Male 12-week-old C57BL/6 (Orient Bio, Gyeonggi, South Korea) and DKK2-Tg mice (provided by Young-Guen Kwon from Yonsei University, Korea.) were used in this study. DKK2-Tg mice express mouse DKK2 under the control of the endothelial cell-specific Tie2 promoter/enhancer. DKK2-Tg mice were backcrossed with C57BL/6 for at least seven generations^17^. All experiments and methods were conducted in accordance with the ethical guidelines and approved by the Institutional Animal Care and Use Committee of Inha University (Assurance Number: INHA 140110-267-1).

To test the efficacy of DKK2 protein, the mice were distributed into three groups: a sham operation group and bilateral CNI groups receiving intracavernous injections of PBS or DKK2 protein (days -3 and 0; 6 µg/20 µl). In the CNI groups, the cavernous nerves were crushed by use of a nonserrated hemostat (Karl Stortz Co., Tuttlingen, Germany). The hemostat was applied with full tip closure to each cavernous nerve 1 mm distal to the ganglion for 2 minutes as previously described^10^. All procedures were done with the aid of a dissecting microscope (Zeiss, Göttingen, Germany). DKK2 protein was administered immediately after CNI. We evaluated erectile function by cavernous nerve electrical stimulation 1 and 2 weeks after treatment.

For the DKK2-Tg study, the mice were distributed into three groups: WT mice receiving sham operation, and WT or DKK-Tg mice receiving bilateral CNI. At 2 weeks after CNI, we measured erectile function during electrical stimulation of the cavernous nerve.

### Measurement of erectile function

The mice from each group were anesthetized with ketamine (100 mg/kg) and xylazine (5 mg/kg) intramuscularly. Bipolar platinum wire electrodes were placed around the cavernous nerve. Stimulation parameters were 5 V at a frequency of 12 Hz, a pulse width of 1 ms, and a duration of 1 min. During tumescence, the maximal ICP was recorded. The total ICP was determined by the area under the curve from the beginning of cavernous nerve stimulation to a point 20 s after stimulus termination. Systemic blood pressure was measured by using a noninvasive tail-cuff system (Visitech systems, Apex, NC, USA). The ratios of maximal ICP and total ICP (area under the curve) to MSBP were calculated to adjust for variations in systemic blood pressure.

### Cell culture experiments

Tube formation assay in primary cultured MCEC and MCP was performed as described in the Supplemental information. PC12 cells (a clonal cell line of rat pheochromocytoma) were cultured in Dulbecco’s modified Eagle Medium supplemented with 10% horse serum, 5% FBS, and 1% penicillin/streptomycin. To mimic an *in vivo* model for neuronal dysfunction or neural degeneration, PC12 cells were exposed to LPS (Sigma-Aldrich, St. Louis, MO, USA; 3 μl/ml) for 24 hours, which were then treated with PBS or DKK2 protein (300 ng/ml).

### *Ex vivo* neurite sprouting assay

Isolation of MPG tissue and *ex vivo* neurite sprouting assay was performed as described in the Supplemental information.

### Histologic examinations

The penis tissue and cultured MPG were fixed in 4% paraformaldehyde for 24 hours at 4°C, and frozen tissue sections (12-μm thick) were incubated with antibodies to nNOS (Santa Cruz Biotechnology, Santa Cruz, CA, USA; 1:50), neurofilament (Sigma-Aldrich; 1: 50), PECAM-1 (Millipore, Temecula, CA, USA; 1:50), NG2 (Millipore; 1:50), cleaved caspase-3 (Novus Biologicals, Littleton, CO, USA; 1:50), or BrdU (Sigma-Aldrich; 1:50) at 4°C overnight. After several washes with PBS, the tissues were incubated with tetramethyl rhodamine isothiocyanate- or fluorescein isothiocyanate-conjugated secondary antibodies (Zymed Laboratories, South San Francisco, CA, USA) for 2 hours at room temperature. Samples were mounted in a solution containing 4,6-diamidino-2-phenylindole (DAPI, Vector Laboratories Inc., Burlingame, CA, USA) for nuclei staining. Signals were visualized and digital images were obtained with a confocal microscope (FV1000, Olympus, Tokyo, Japan). For 5′-bromo-2′-deoxyuridine (BrdU) labeling experiments, an additional antigen retrieval step was performed. Briefly, following fixation, the sections were washed with PBS three times and incubated for 10 min in 1 M HCl on ice and for 20 min in 2 M HCl at 37 °C to allow denaturation of DNA. The sections were then given three 5-minute washes in 0.1 M sodium borate buffer (Na_2_B_4_O_7_). Quantitative analysis of histologic examinations was done with an image analyzer system (National Institutes of Health [NIH] Image J 1.34, http://rsbweb.nih.gov/ij/).

### BrdU labeling

The mice from each group were given intraperitoneal injections of BrdU (Sigma-Aldrich; 50 mg/kg of body weight) once a day for three consecutive days and were sacrificed one day after BrdU injection. The number of BrdU-positive endothelial cells was counted at a screen magnification of ×400 in 6 or 8 different regions. Values were expressed per high-power field.

### Western blot

Equal amounts of protein (20 µg per lane) were electrophoresed on sodium dodecylsulfate-polyacrylamide gels (12% to 15%), transferred to nitrocellulose membranes, and probed with antibodies to NGF (Santa Cruz Biotechnology; 1:1000), BDNF (Santa Cruz Biotechnology; 1:1000), NT-3 (Santa Cruz Biotechnology; 1:1000), GAPDH (ABclonal, Woburn, MA, USA; 1:5000), or β-actin (Abcam; 1:6000). The results were quantified by densitometry.

### Statistical analysis

The results are expressed as mean ± SE. For parametric data, intergroup comparisons were made by one-way ANOVA followed by Newman-Keuls post-hoc tests. We used the Mann-Whitney *U* test or Kruskal-Wallis test to compare nonparametric data. Probability values less than 5% were considered significant. We used SigmaStat 3.11 software (Systat Software) for statistical analyses.

## Electronic supplementary material

Supplemental Information
