# Supplementary material for: Dickkopf2 rescues erectile function by enhancing penile neurovascular regeneration in a mouse model of cavernous nerve injury
Source: Sci Rep. 2017 Dec 19;7:17819. doi: 10.1038/s41598-017-17862-5 (PMC5736639; doi:10.1038/s41598-017-17862-5)
Supplement: Supplementary file 1 — Supplemental Information [file 41598_2017_17862_MOESM1_ESM.doc]

#### **Supplementary Information**

#### **Dickkopf2 rescues erectile function by enhancing penile neurovascular regeneration in a mouse model of cavernous nerve injury**

Kalyan Ghatak^1,*^, Guo Nan Yin^1,*^, Min-Ji Choi^1^, Anita Limanjaya^1^, Nguyen Nhat Minh^1^, Jiyeon Ock^1^, Kang-Moon Song^1^, Dong Hyuk Kang^1^, Young-Guen Kwon^2^, Ho Min Kim^3^, Ji-Kan Ryu^1,4, †^, Jun-Kyu Suh^1,†^

^1^National Research Center for Sexual Medicine and Department of Urology, Inha University School of Medicine, Incheon 22332, Republic of Korea

^2^Department of Biochemistry, College of Life Science and Biotechnology, Yonsei University, Seoul 03722, Republic of Korea

^3^Graduate School of Medical Science and Engineering, Korea Advanced Institute of Science and Technology (KAIST), Daejeon 34141, Republic of Korea

^4^**Inha Research Institute for Medical Sciences,** Inha University College of Medicine, **Incheon** 22212**,** Republic of Korea

^*^Kalyan Ghatak and Guo Nan Yin contributed equally to this study.

^†^Correspondence to Ji-Kan Ryu, MD, PhD or Jun-Kyu Suh, MD, PhD

Ji-Kan Ryu, MD, PhD

National Research Center for Sexual Medicine and Department of Urology, **Inha Research Institute for Medical Sciences**

Inha University College of Medicine

7-206, 3^rd^ ST, Shinheung-Dong, Jung-Gu, Incheon 22332

Republic of Korea

Tel: 82-32-890-3505; Fax: 82-32-890-3099

E-mail: rjk0929@inha.ac.kr

Jun-Kyu Suh, MD, PhD

National Research Center for Sexual Medicine and Department of Urology

Inha University College of Medicine

7-206, 3^rd^ St, Shinheung-Dong, Jung-Gu

Incheon 22332, Republic of Korea

Tel: 82-32-890-3441, Fax: 82-32-890-3097

E-mail: jksuh@inha.ac.kr

**Supplementary Methods**

**Cell culture experiments**

The mouse cavernous endothelial cells (MCEC) and mouse cavernous pericytes (MCP) were prepared and maintained as previously described^1-3^. Penis tissue was harvested and transferred into sterile vials containing Hank’s balanced salt solution (GIBCO, Carlsbad, CA, USA) and was washed two times in PBS. The glans penis, urethra, and dorsal neurovascular bundle were removed from the penis, and only the corpus cavernosum tissue was used for primary endothelial cell culture.

For MCEC culture, the corpus cavernosum tissue was cut into two or three pieces and the samples were then plated on matrigel (Becton Dickinson, Mountain View, CA, USA)-coated 60-mm cell culture dishes. The matrigel was polymerized with a 5-min incubation period at 37°C, and 3 ml of complement medium 199 (GIBCO), supplemented with 20% fetal bovine serum (FBS), 1% penicillin/ streptomycin, 0.5 mg/ml heparin, and 5 ng/ml vascular endothelial growth factor, was added to the cell culture dish, and the dishes were then incubated at 37°C with 5% CO_2_. After the cells were confluent and spread on the whole bottom of the dish (about 2 to 3 weeks after the start of culture), only sprouting cells were used for subcultivation. The sprouting cells were seeded onto dishes coated with 0.2% gelatin (Sigma-Aldrich, St. Louis, MO, USA).

For MCP culture, the corpus cavernosum tissue was cut into several pieces (1 mm) and the fragmented pieces of cavernous samples were settled by gravity into collagen I-coated 35-mm cell culture dishes (BD Biosciences, San Jose, CA, USA). After 30 minutes incubation at 37°C with 300 µl complement Dulbecco’s modified Eagle Medium (DMEM, GIBCO), supplemented with 10% FBS, 1% penicillin/streptomycin, and 10 nM human pigment epithelium-derived factor (PEDF; Sigma-Aldrich), we added 900 µl complement medium additionally and the samples were incubated at 37°C with 5% CO_2_. The medium was changed each 2 days. After the cells were confluent and spread on the whole bottom of the dish (about 2 weeks after the start of culture), only sprouting cells were used for subcultivation. The sprouting cells were seeded onto dishes coated with 50 µl/ml collagen I (Advanced BioMatrix, San Diego, CA, USA). Cells at passages between 2 and 3 were used for experiments.

**Establishment of MCEC-MCP co-culture system**

We established endothelial cell-pericyte co-culture system by using primary cultured MCEC and MCP as previously described^4^. To determine optimal ratio of MCEC and MCP for direct mixed co-culture system, the cells were mixed at different ratio (MCEC: MCP = 10:1, 5:1, 3:1, 2:1, 1:1) and cultivated in 50% DMEM and 50% M199 complement medium. The mixture of MCEC and MCP formed well-organized capillary-like structures at the ratio of 3:1^4^. Based on our previous result, the MCEC and MCP were cultivated at the ratio of 3:1 for *in vitro* angiogenesis assay.

***In vitro* angiogenesis assay**

The tube formation assay was performed to assess the angiogenic capacity of DKK2 protein in MCEC or MCP. About 50 µl of growth factor-reduced matrigel was dispensed into 96-well tissue culture plates at 4°C. After gelling at 37°C for at least 30 min, the MCEC or MCP were seeded onto the gel at 2 × 10^4^ cells/well in 200 µl of M199 or DMEM medium. The assay was performed in a CO_2_ incubator and the plates were incubated at 37°C for 24 hours. Images were obtained with a phase-contrast microscope and the numbers of tubes in each well of the plate were counted at a screen magnification of ×40. Only integrated tubes were counted.

***Ex vivo* neurite sprouting assay**

The mouse MPG tissues were prepared and maintained as previously described^5^ with minor modifications. The MPG tissues were isolated from male mouse by the use of microscope and transferred into sterile vials containing Hank’s balanced salt solution (GIBCO) and then rinsed and washed twice in PBS. The MPG tissues were cut into small pieces and the samples were then plated on poly-D-lysine hydrobromide (Sigma-Aldrich)-coated 12-well plate. The whole MPG tissues were covered with matrigel and the culture plate was placed on ice for 5 min and then incubated at 37°C for 10-15 min with 5% CO_2_. We added 1 ml of complete Neurobasal Medium (GIBCO), supplemented with 2% serum free B-27 (GIBCO) and 0.5 nM GlutaMAX™-I (GIBCO). The dishes were then incubated at 37°C with 5% CO_2_. At 5 days after incubation, we evaluated neurite outgrowth.

**References**

1. Yin, G.N. *et al.* The pericyte as a cellular regulator of penile erection and a novel therapeutic target for erectile dysfunction. *Sci. Rep.* **5**, 10891 (2015).

2. Neng, L. *et al.* Isolation and culture of endothelial cells, pericytes and perivascular resident macrophage-like melanocytes from the young mouse ear. *Nat. Protoc.* **8**, 709-720 (2013).

3. Yin, G.N. *et al.* Matrigel-based sprouting endothelial cell culture system from mouse corpus cavernosum is potentially useful for the study of endothelial and erectile dysfunction related to high-glucose exposure. *J. Sex. Med.* **9**, 1760-1772 (2012).

4. Yin, G.N. *et al*. Establishment of in vitro model of erectile dysfunction for the study of high-glucose-induced angiopathy and neuropathy. *Andrology* 2016 Dec 19. doi: 10.1111/andr.12307. [Epub ahead of print]

5. Lin, G. *et al.* Neurotrophic effects of vascular endothelial growth factor and neurotrophins on cultured major pelvic ganglia. *BJU Int.* **92**, 631-635 (2003).

**Supplementary Figure S1**


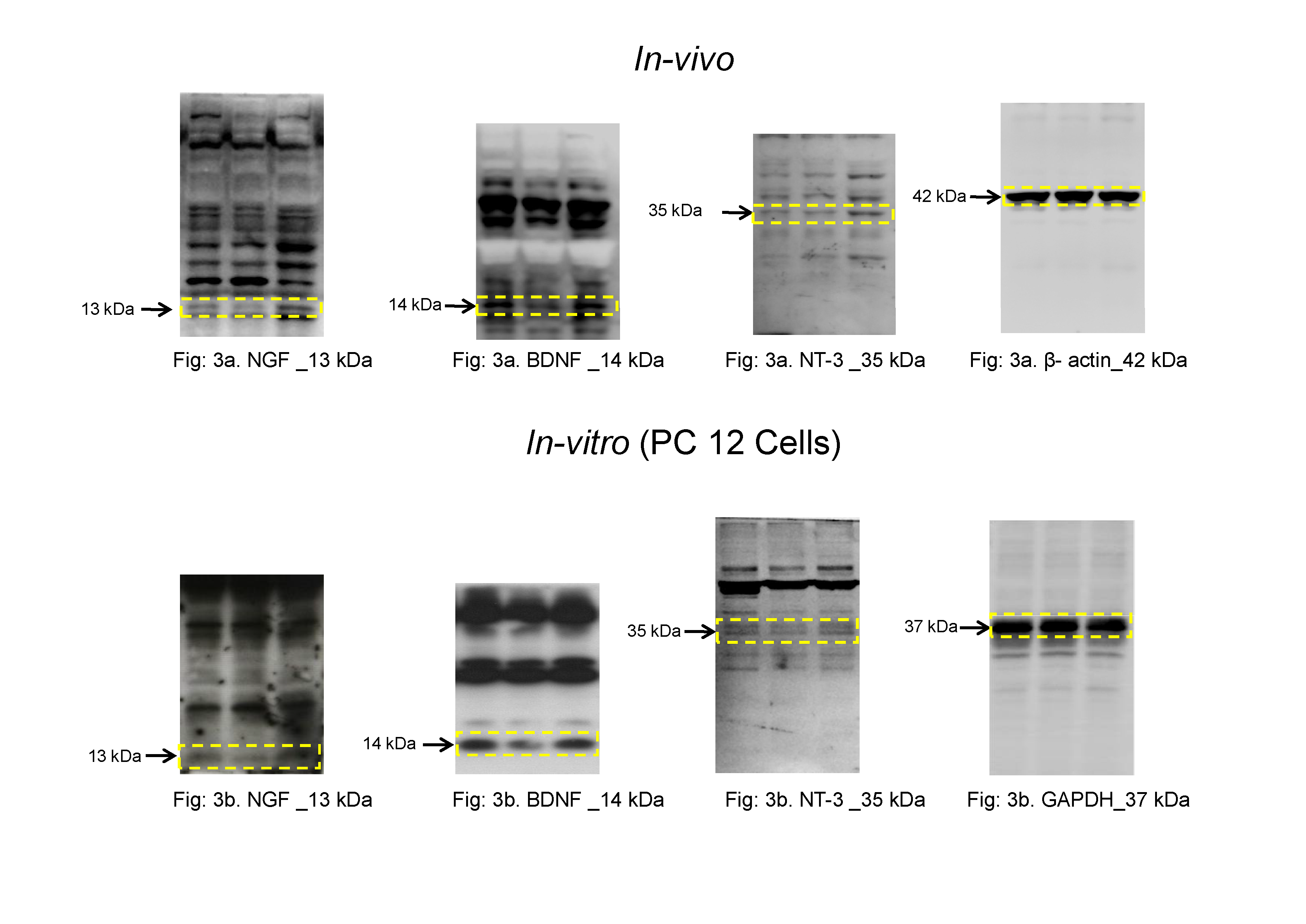


**Supplementary Figure S1.** Full-length blots/gels images for neurotrophic factors.

**Supplementary Tables**

Table S1 Physiological parameters 1 or 2 weeks after cavernous nerve injury

|  | Cavernous nerve injury | | | | | |
| --- | --- | --- | --- | --- | --- | --- |
|  | 1week | | | 2 weeks | | |
|  | Sham | PBS | DKK2 | Sham | PBS | DKK2 |
| Body weight(g) | 24.5±0.44 | 24.9±0.42 | 24.6±0.45 | 25.8±0.39 | 26.2±0.79 | 25.2±0.47 |
| MSBP | 86.5±1.4 | 98.7±1.9 | 91.2±1.5 | 89.0±1.4 | 85.0±1.6 | 92.0±1.3 |

Values are the mean ± standard error from n = 6 animals per group. MSBP = mean systolic blood pressure; PBS = phosphate-buffrered saline

Table S2 Physiological parameters 2 weeks after cavernous nerve injury

|  | Cavernous nerve injury | | |
| --- | --- | --- | --- |
|  | Sham | WT | DKK2-Tg |
| Body weight (g) | 26.1±0.33 | 25.8±0.32 | 26.4±0.32 |
| MSBP | 86.5±1.4 | 95.7±2.1 | 88.2±2.2 |

Values are the mean ± standard error from n = 4 animals per group. MSBP = mean systolic blood pressure; PBS = phosphate-buffrered saline; DKK2-Tg= Dickkopf2 transgenic mice
